# A comparative study of the efficacy and safety of radiofrequency ablation and botulinum toxin A in treating masseteric hypertrophy

**DOI:** 10.3892/etm.2014.1552

**Published:** 2014-02-17

**Authors:** JIN-LONG HUANG, GANG CHEN, XIAO-DONG CHEN, BING-RONG ZHOU, DAN LUO

**Affiliations:** 1 Department of Esthetic Plastic Surgery, The First Affiliated Hospital of Nanjing University of TCM, Nanjing, Jiangsu 210029, P.R. China; 2 Department of Dermatology, The First Affiliated Hospital of Nanjing Medical University, Nanjing, Jiangsu 210029, P.R. China

**Keywords:** masseteric hypertrophy, botulinum toxin A, radiofrequency ablation

## Abstract

The aim of the present study was to evaluate the effects of two treatments for masseteric hypertrophy. In total, 24 patients with masseteric hypertrophy were enrolled in this study. Patients were randomly divided into two groups: 12 individuals were treated with radiofrequency (RF) ablation and 12 patients received an injection of botulinum toxin A. The thickness of the masseter muscle under tension was measured using ultrasound and clinical photographs were captured prior to treatment and at 6 and 12 months following treatment. Complications were observed during 12-month follow-up. In the group injected with botulinum toxin A, masseteric muscle thickness decreased to the lowest point 6 months after the injections but increased until 12 months after injection. However, in the group treated with RF ablation, muscle thickness decreased steadily over the 12 months following surgery. Therefore, the results of the present study indicated that the effect of RF ablation on the thickness of the masseter muscle may be much larger than that obtained following injection with botulinum toxin A.

## Introduction

Benign masseteric hypertrophy is a major reason for a wide lower face. Several treatment methods may be used on patients with masseteric hypertrophy in order to improve facial contours. To date, the major treatment methods involve surgery, including mandibular angle osteotomy and masseter muscle resection, and there are non-surgical methods, including injection with botulinum toxin type A (1,2). Jin Park *et al* reported on radiofrequency (RF) volumetric reduction for masseteric hypertrophy (3). RF-induced tissue coagulation necrosis of the masseter muscle did not lead to any infections or limitations with regard to mouth opening, and the clinical improvement was well maintained following treatment (3). However, thus far, there have been no studies on the efficacy and safety of RF and injection with botulinum toxin A for the treatment of masseteric hypertrophy. In the present study, these two methods were compared by measuring the thickness of the masseter muscle at 6 and 12 months following surgery.

## Materials and methods

### Patients

The study was approved by the Institutional Review Board of the Affiliated Hospital of Nanjing University of Traditional Chinese Medicine (Nanjing, China). A total of 24 Chinese patients (female, 23; male, 1; mean age, 27 years; age range, 21–48 years; Fitzpatrick skin types, IV) with moderate to severe masseteric hypertrophy were included in this study. All patients provided informed consent and were randomly assigned to two groups (the botulinum toxin A and RF ablation groups). Patients with concomitant treatments, including surgical resection or RF ablation of the masseter muscle nerve and injection with botulinum toxin A into the muscle in the previous year, were excluded. Patients who were pregnant or who presented with a neuromuscular or immunosuppressive disease were also excluded from this study.

### Botulinum toxin A injection

The masseter muscle on both sides was treated with botulinum toxin A (Lanzhou Biological Products Institute of China, Lanzhou, China) at three identical injection sites. After asking the patients to clench their teeth, the anterior and posterior borders of the master muscle were identified. The injection points were below the ear lobe-mouth corner line and ~1.5 cm above the mandibular angle border. The injection points were in the center of the lower third of the muscle, 1 cm apart. Injections were perpendicular to the skin and intramuscular to the middle third of the needle. The doses of botulinum toxin A were determined according to the individual severity of masseteric hypertrophy. Photographs were captured prior to and following the injections at the baseline and at 2, 8 and 12 weeks following treatment.

### RF ablation treatment

Prior to surgery, the masseter muscle margins were marked with gloss vinyl ink while the patients clenched their jaws. Subsequently, diagrams for the insertion of the RF probes were created by drawing several parallel lines on the skin within the lower third of the masseter muscles. In addition, four to five horizontal parallel lines and two oblique lines were marked on the masseter muscles. Surgery was performed in an outpatient room with the patients under local anesthesia and sedation. All the patients received 5 mg valium 30 min prior to the procedure for its sedative effect. Once the patients had gargled with a povidone solution, a 10% xylocaine spray was applied to the oral mucosa. Subsequently, 3.6 ml lidocaine (1%) with 1:100,000 epinephrine solution was injected evenly into the masseter muscles through the oral mucosa. Next, 7-mm-long exposed active tips of 120-mm-long insulated needle electrodes were placed into the masseter muscles. A 17-gauge guide needle and custom-designed monopolar RF probes (Ellman International, Inc., New York, NY, USA) were inserted into the lower third of the masseter muscles, as close to the periosteum of mandible as possible, in order to prevent any thermal injury to the adjacent soft tissues, buccal branches of the facial nerves, deep middle masseteric arteries or Stensen’s ducts. A protective sheath was used on the proximal portions of the electrodes to diminish any injury to the oral mucosa. The probes were located slightly below ~30 mm above the gonion, along the posterior border of the masseter muscles in order to prevent damage to the deep middle masseteric arteries, which enter the masseter muscles from the external carotid arteries ~30 mm above the gonion. RF energy (mean power, 40 W±10%) was delivered with monopolar electrodes over a period of 5–10 sec for each spot.

### Evaluation

To evaluate the long-term clinical effects, any side effects were monitored during follow-up. Ultrasound measurements of the thickness of the masseter muscle under tension were performed and clinical photographs were captured prior to treatment and at 6 and 12 months following treatment.

### Statistical analysis

Data were analyzed using SPSS 11.0 software (SPPS, Inc., Chicago, IL, USA). The statistical significance of the difference between the groups was determined with one way analysis of variance followed by the Bonferroni t-test for multiple comparisons. P<0.05 was considered to indicate a statistically significant difference.

## Results

### Masseteric thickness

**Figure 1:**
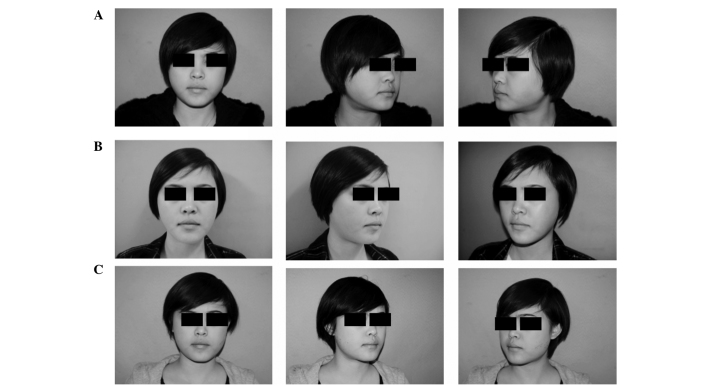
A 21-year-old female treated with an injection of botulinum toxin A. Imaged captured (A) preoperatively, (B) 6 months following treatment and (C) 12 months following treatment.

**Figure 2:**
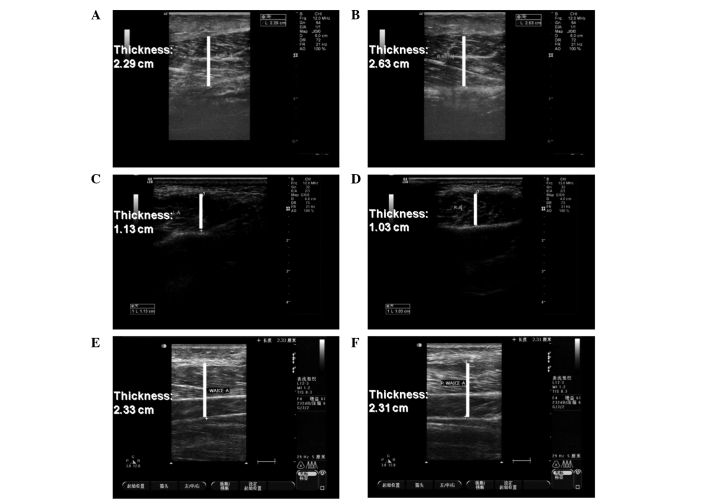
Ultrasound evaluation of the right and left sides of the patient shown in Fig. 2 (A and B) prior to treatment, (C and D) 6 months after treatment and (E and F) 12 months after treatment. The white bar indicates the thickness of the masseter muscle.

**Figure 3:**
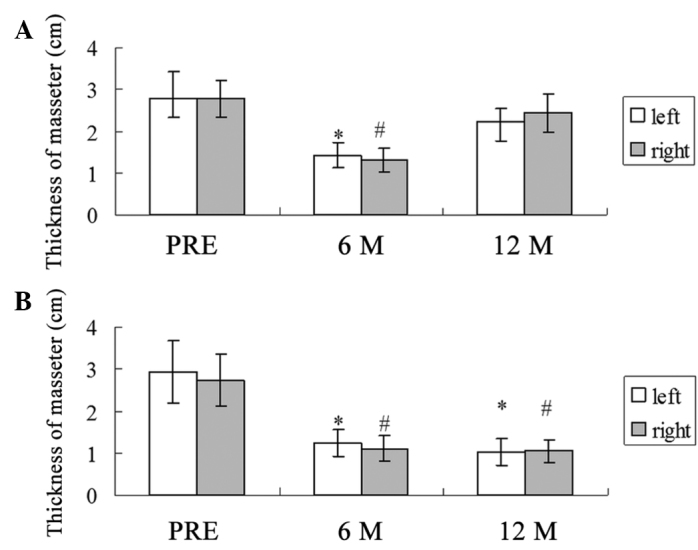
Comparing the thickness of the masseter muscle pre- and post-treatment in (A) RF ablation and (B) botulinum toxin A injection groups. ^*^P<0.05 (left side) and ^#^P<0.05 (right side), vs. pre-treatment. PRE, pre-treatment; 6 M, 6 months following treatment; 12 M, 12 months following treatment; RF, radiofrequency.

**Figure 4:**
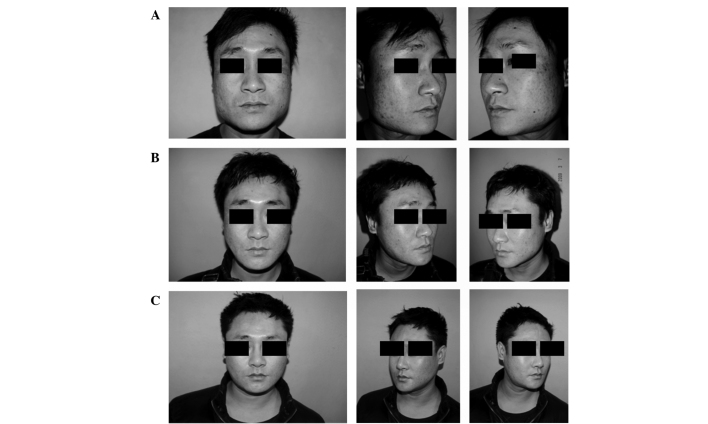
A 25-year-old male treated by RF ablation. Images captured (A) preoperatively, (B) 6 months following treatment and (C) 12 months following treatment. The bulky lower facial contour was markedly reduced. RF, radiofrequency.

**Figure 5:**
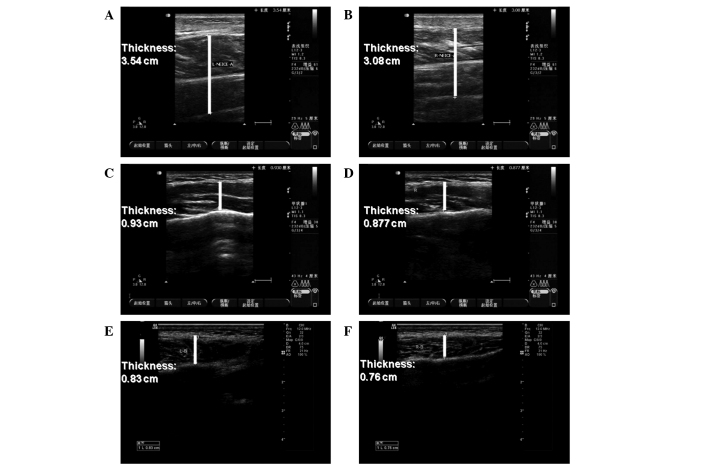
Ultrasound evaluation of the right and left sides of the patient shown in Fig. 1 (A and B) prior to treatment, (C and D) 6 months following treatment and (E and F) 12 months after treatment. The white bar indicates the thickness of the masseter muscle.

The mean masseteric thicknesses prior to injection with botulinum toxin A were 2.79 cm on the left side and 2.78 cm on the right side, as measured by ultrasound. The mean masseteric thicknesses at 6 and 12 months after injection were 1.43 and 1.31 cm on the left side, and 2.24 and 2.45 cm on the right side, respectively (Figs. 1, 2 and 3A). The mean masseteric thicknesses prior to treatment with RF ablation were 2.93 cm on the left side and 2.73 cm on the right side. The mean masseteric thicknesses at 6 and 12 months following surgery were 1.23 and 1.11 mm on the left side, and 1.02 and 1.05 cm on the right side, respectively (Figs. 3B, 4 and 5). In the botulinum toxin A injection group, the masseteric thickness measured by ultrasound decreased to the lowest point 6 months after the injections, but increased 12 months following the injection. However, in the RF ablation treatment group, masseteric thickness decreased steadily over the 12 months following surgery (Figs. 2, 4 and 5).

### Complications

**Figure 6:**
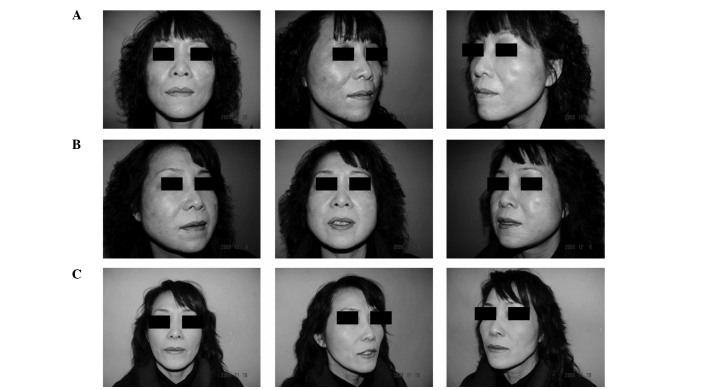
A 45-year-old female treated by RF ablation. Images captured (A) preoperatively, (B) 1 week after surgery. (C) 12 months following surgery. RF, radiofrequency.

In the botulinum toxin A injection group, the main local side effects were difficulty in masticating hard food types, speech disturbance and pain at the injection sites. These complaints were transient, usually lasting between 1 and 4 weeks after the injection. Furthermore, facial asymmetry and prominent zygoma were observed. For those treated with RF ablation, marked swelling was observed in the treatment area within 1–2 weeks, but disappeared two weeks later (Fig. 6). One patient demonstrated unilateral intraoperative district hematoma 3 days after surgery. Pressure was applied with bandages to the hematoma area for 3 days and the hematoma diminished 2 months later. One patient exhibited little effect 2 months after the surgery and received a second ablation. Following the second ablation, the patient was satisfied with the effect. All patients complained of discomfort while chewing and mouth pain of varying degrees within 2 weeks of the surgery, however, these discomforts disappeared spontaneously. No patients complained of injuries to the facial nerve or of parotid duct or oral dysfunction.

## Discussion

Masseteric hypertrophy can be treated with surgical or non-surgical methods (4,5). It is difficult to assess the amount and depth of resection of the masseter muscle during surgical excision. Furthermore, the process is complex and may result in postoperative complications, including bleeding, hematoma, facial nerve damage, asymmetry and the inability to masticate (2,4). Botulinum toxin A injection for the treatment of masseteric hypertrophy is popular due to the simplicity of the procedure and the rapid postoperative recovery times (6). In 2007, Jin Park *et al* first reported on the usage of RF ablation for the correction of masseteric hypertrophy in 340 patients and achieved favorable clinical results (3). To date, there have been no studies comparing the treatment and side effects of injection with botulinum toxin A with those of RF ablation for the treatment of masseteric hypertrophy.

In the present study, the thickness of the masseter muscle was analyzed pre- and post-botulinum toxin A injection or RF ablation treatment in 24 Chinese patients. In the botulinum toxin A injection group, masseteric thickness decreased to the lowest point 6 months after the injection, but increased until 12 months after the injection. However, in the RF ablation treatment group, muscle thickness decreased steadily over the 12 months following surgery.

The main advantages of injection with botulinum toxin A when treating masseteric hypertrophy are that the procedure is easy with fast postoperative recovery (7). Toxin injection was demonstrated to be safe with no evident complications occurring within two years of the injection. However, the present study indicated that the efficacy of botulinum toxin A lasted for a shorter time when compared with RF ablation. Within 6 months of the injection, the majority of the participants reported an improvement in their facial contours. However, in the botulinum toxin A injection treatment group, the thickness returned to the baseline 12 months after the injection. Therefore, in order to maintain the therapeutic effects, the patients usually require repeated injections.

As demonstrated by the results of the present study, RF ablation exhibited no serious complications for the treatment of masseteric hypertrophy. However, a previous study has reported that a mild injury to the facial nerve and parotid duct may occur postoperatively (3). In order to avoid parotid duct and facial nerve injury, in the present study, a new needle electrode was designed that reduced the amount of mucosal injury. One patient was unsatisfied with the postoperative results after receiving RF ablation treatment, which may be due to the lack of experience when using this newly designed RF ablation setting. In addition, when assessing the clinical experiences, the shortcomings of RF ablation included clear swelling 1–2 weeks following surgery and certain patients were unable to withstand this therapy. In order to achieve the expected results for the change in masseter muscle volume, locating an accurate anatomical location of the masseter muscle is required, as well as having a good understanding of the performance of the instrument. However, there are certain advantages to RF ablation therapy when compared with other therapies. Firstly, the patient may eat after surgery. Secondly, the biting force of the teeth returns to normal within 3 months of the surgery. Thirdly, 3 months after treatment, the masseter muscle retains the shrinking trend and the effect appears stable even at 6 months following surgery. Finally, RF therapy carries a low risk of injury to the peripheral structures. Therefore, it may be concluded that injection with botulinum toxin A and RF ablation therapy are safe and effective treatment options for masseteric hypertrophy.

The present study had certain limitations. A split-face study was not conducted on a patient when comparing these two methods. However, very few patients are willing to accept such treatments. Furthermore, as this is a single-center study, the observations of the present study require further verification in a study with a larger number of patients.
